# Alpha-Fetoprotein as a Predictive Marker for Patients with Hepatitis B-Related Acute-on-Chronic Liver Failure

**DOI:** 10.1155/2018/1232785

**Published:** 2018-05-09

**Authors:** Xiaoping Wang, Caifei Shen, Jianjiang Yang, Xianjun Yang, Sen Qin, Haijun Zeng, Xiaoling Wu, Shanhong Tang, Weizheng Zeng

**Affiliations:** ^1^Department of Gastroenterology, Chengdu Military General Hospital, Chengdu, Sichuan, China; ^2^College of Medicine, Southwest Jiaotong University, Chengdu, Sichuan, China; ^3^Chengdu Military Command Disease Prevention and Control Center, Chengdu, Sichuan, China

## Abstract

**Background and Aims:**

The value of alpha-fetoprotein (AFP) in hepatitis B-related acute-on-chronic liver failure (HBACLF) is not fully understood. The present study aimed to evaluate the prognostic effect of AFP on the prediction of HBACLF outcomes.

**Methods:**

We investigated a cohort of patients with HBACLF admitted from January 2013 to May 2017. The endpoint of followup was 180 days, death, or liver transplantation. AFP concentrations were estimated on admission. To make statistical comparisons, we used chi-squared test, receiver operating characteristic (ROC) curve analysis, survivorship curve analysis, and Cox proportional-hazards model.

**Results:**

A total of 92 patients (81.5% male, median age of 46 years) were included. Overall survival rate within 180 days was 43.48%, and the value of log_10_^AFP^⁡  ≥ 2.04 indicated a better prognosis with 76.9% specificity and 62.5% sensitivity for patients with HBACLF. Age (HR 1.041), total bilirubin (HR 1.004), log_10_^AFP^⁡  (HR 2.155), and INR (HR 1.446) were found to be risk factors of survival.

**Conclusion:**

AFP could be a useful marker to predict outcomes of acute-on-chronic liver failure.

## 1. Introduction

Acute-on-chronic liver failure (ACLF) is defined by a rapid progression in hepatic dysfunction induced by certain precipitating events due to previous liver diseases, resulting in multisystem organ failure and high short-term mortality [1]. There is no specific treatment for ACLF, and the most effective therapy is liver transplantation. However, the shortage of liver donations has largely hindered its wide implementation. Supporting the regeneration of hepatocytes and preventing the complications tend to decrease the mortality of ACLF, and artificial liver support is a useful method to manage ACLF [2]. The aetiologies of ACLF vary between territories. Viral infections are more common in Asia, while there is a wide prevalence of alcoholic cirrhosis and nonalcoholic fatty liver in American and European countries [3–6]. With a carrier rate of hepatitis B virus (HBV) surface antigen at approximately 8% in adults, China exhibits a high morbidity of hepatitis B, which is the most common aetiological factor of ACLF [7].

Some scoring systems efficiently assess the severity and mortality of ACLF, such as SOFA score [8], MELD [9], and King's College Criteria [10]. Other parameters including bilirubin and international normalized ratio (INR) are treated as prognostic markers in clinical practice as well. Those methods primarily focus on the impaired hepatic function, while seldom did researchers concentrate on parameters of hepatocyte regeneration and evaluate their prognostic value for ACLF. Typically, AFP is the most abundant plasma protein in foetuses, and serum AFP remains elevated in infant livers until several weeks after birth. High serum AFP expression in adults generally indicates a high possibility of hepatocellular carcinoma in patients with chronic hepatitis or cirrhosis [11]. Meanwhile, AFP is considered a biomarker of proliferating liver stem cells in liver injury conditions as well, and the recruitment of liver progenitor cells is associated with a better outcome for liver failure [12, 13].

The discovery of AFP dates back to 1960s. Since then, AFP has been investigated in the field of liver diseases. Previous studies demonstrated that AFP was a vital prognostic marker for outcomes in patients with acute liver failure [14–16]. However, few studies evaluated the outcomes of ACLF from the perspective of hepatocyte regeneration. As we know, ACLF exhibits a small window during which liver dysfunction may be reversed, and the repair of liver tissues is tightly correlated with hepatocyte regeneration, which presents as increased AFP levels. Therefore, we performed a retrospective study to identify whether AFP was a valid predictive indicator of outcomes in ACLF patients.

## 2. Materials and Methods

### 2.1. Study Population

**Figure 1:**
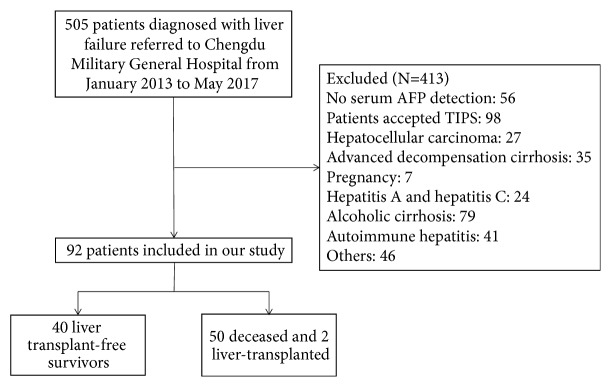
Inclusion and exclusion criteria for the study.

We concentrated on a cohort of patients with hepatitis B-related acute-on-chronic liver failure (HBACLF). A total of 505 patients with suspected ACLF were enrolled in our study from January 2013 to May 2017 at Chengdu Military General Hospital, Sichuan, China. Researchers who undertook the work of selecting cases as the main subjects were blind to the serum AFP concentrations of those patients. ACLF is diagnosed according to the criteria of Asian Pacific Association for the Study of the Liver (APASL): serum bilirubin ≥ 85 *μ*mol/L, INR ≥ 1.5 or prothrombin activity ≤ 40%, any degree of encephalopathy and/or clinical ascites within 4 weeks, and an evidence of ongoing chronic liver diseases [3]. Patients who were diagnosed with ACLF and aged 18 to 75 years were included. A total of 413 patients in our database were excluded for the following reasons: (1) lack of serum AFP concentrations; (2) manifestation of decompensated liver cirrhosis prior to ACLF diagnosis, such as ascites and variceal haemorrhage; (3) patients with portal hypertension who received a transjugular intrahepatic portosystemic shunt (TIPS); (4) patients pathologically diagnosed with or clinically suspected for hepatocellular carcinoma; (5) other malignancies such as gastric cancer; (6) pregnancy; (7) HIV or hepatotropic virus infection other than HBV; and (8) other preexisting chronic liver diseases, such as fatty liver, autoimmune hepatitis, and alcoholic cirrhosis (Figure 1). The final cohort contained 92 patients with a median age of 46 years (range, 18–75 years), and all patients received antiviral therapy by orally taking tenofovir or entecavir. Besides, reduced glutathione was given to protect the liver from subsequent damage.

### 2.2. Clinical and Biological Parameters

The clinical parameters included ascites and hepatic encephalopathy (HE). The biological parameters included AFP, INR, and total bilirubin. The serum AFP levels were measured on admission.

### 2.3. Followup

The end point of observation was 180 days, death, or liver transplantation. Forty of the 92 patients survived spontaneously, 50 patients died, and 2 patients received liver transplantation.

### 2.4. Statistical Analysis

Results are presented as means and standard deviations (SDs) and median and range appropriately. The chi-squared test was used to compare rates between groups. Receiver operating characteristic (ROC) curve analysis was performed. Survival was estimated by Kaplan-Meier method, and differences were evaluated with log-rank test. Cox proportional-hazards model was adopted to estimate the risk factors of survival. Data were analyzed using SPSS version 16.0 software (IBM Corporation, Somers, NY, USA). Differences were considered to be of statistical significance when the *P* value ≤ 0.05.

## 3. Results

### 3.1. Baseline Characteristics

Ninety-two patients were incorporated in our study, including 17 women (18.5%). The population was divided into two groups based on the prognosis of ACLF. In total, there were 40 liver transplant-free survivors, 50 deceased patients, and 2 liver-transplanted patients.  Table 1 depicts the demographic and biochemical characters of the two groups. Age, total bilirubin, AFP, and INR differed significantly between transplant-free survivors and those who deceased or got liver-transplanted.

### 3.2. AFP as a Predictor for Prognosis of HBACLF

**Figure 2:**
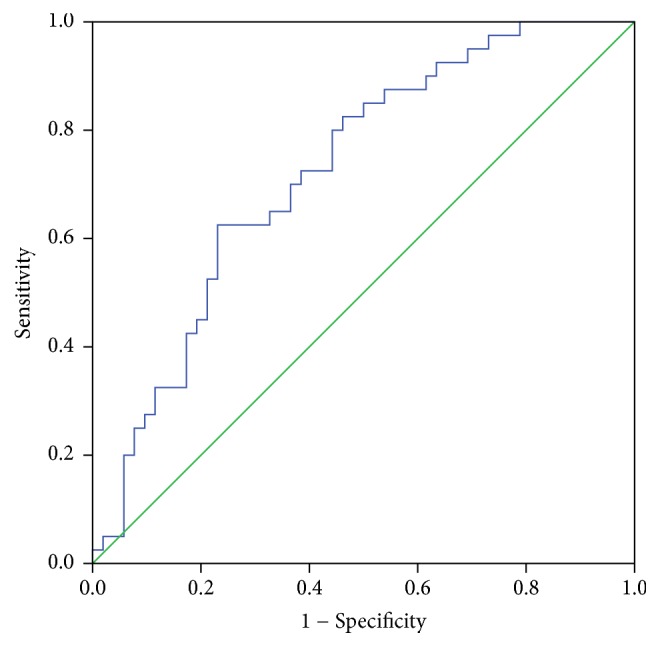
ROC curve for log_10_^AFP^⁡  in predicting the outcome of HBACLF (*n* = 92).

The recruitment of functional hepatocytes is the key to the recovery of the impaired liver function. To estimate the outcome of ACLF from the perspective of hepatocyte regeneration, we evaluated the predictive value of AFP by creating an equation, namely, log_10_^AFP^⁡ , to assess the prognosis of HBACLF. A receiver operating characteristic curve was created for this parameter to predict the outcome of ACLF patients. The area under the curve was 0.725. A cut-off point of log_10_^AFP^⁡  ≥ 2.04 was suggested to indicate a better outcome with 76.9% specificity and 62.5% sensitivity (Figure 2).

### 3.3. log_10_^AFP^⁡  Is a Risk Factor of Survival for HBACLF

**Figure 3:**
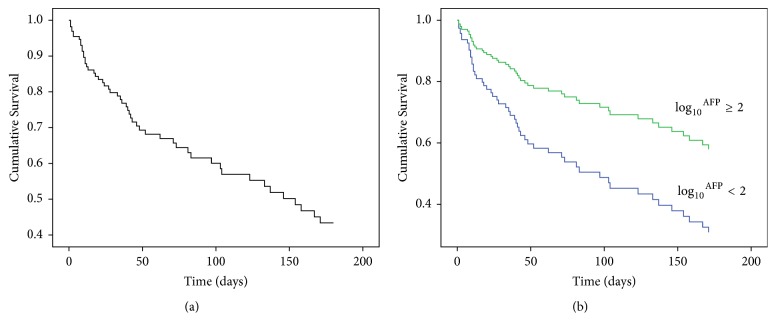
(a) Graph of multivariate Cox regression survival curve of the total population (*n* = 92). (b) Graph of multivariate Cox regression survival curve for patients with log_10_^AFP^⁡  ≥ 2 (*n* = 41) and log_10_^AFP^⁡  < 2 (*n* = 51).

Patients with chronic hepatitis B-related ACLF exhibited an incredibly high mortality within 180 days in our observation. The transplant-free survival rate at 30 days was 72.83%, and it gradually declined to 43.48% at 180 days of followup (Figure 3(a)). Given that patients would probably have a better outcome when log_10_^AFP^⁡  was approximately higher than 2, we then chose log_10_^AFP^⁡  as a categorical variable to assess the survival of HBACLF. In addition, we also adopt demographic parameters and serum biochemical parameters which were considered as representatives of the hepatic function. By using Cox proportional-hazards model, log_10_^AFP^⁡  was found to be an independent factor of survival as a categorical variable, as with continuous variables including age, INR, and levels of total bilirubin (Table 2). There were 41 patients whose log_10_^AFP^⁡  ≥ 2, and this group of patients was more likely to exhibit a longer survival time. The transplant-free survival rates at 30, 90, and 180 days were 58.82% versus 90.24% (*P* = 0.001), 39.22% versus 83.93% (*P* < 0.001), and 29.41% versus 58.54% (*P* = 0.005), respectively, in groups of patients with log_10_^AFP^⁡  < 2 and ≥2 (Figure 3(b)).

## 4. Discussion

The idea of ACLF was first proposed to describe the acute liver damage of an ongoing chronic liver disease in 1995 [17]. There are more than 13 different definitions of ACLF worldwide, which vary from the West to the East. An acute insult may lead to rapid and progressive liver failure in patients with chronic liver diseases and result in high short-term mortality of approximately 50–90% because of the limited functional reserve of the liver [1, 18].

China exhibits a high morbidity of hepatitis B and HBACLF. There are studies estimating the severity and outcomes of HBACLF by establishing prognostic scoring models. Previous studies illustrated that the liver volume [19], lymphocyte-monocyte ratio [20], albumin-bilirubin score [21], logistic regression model [22], macrophage inflammatory protein 3*α* [23], and other methods [24–26] could be simple and sensitive models to evaluate the severity and prognosis of ACLF, which would be practically useful in clinic. Those methods mainly concentrated on the severity of liver injury, and the parameters in those models were generally to reflect the condition of liver function. It is known to us that the prognosis of ACLF depends on the extent of liver injury, the capability of hepatocyte regeneration, and the prevention of multiple organ failure. There are a number of studies evaluating the prognosis of ACLF from the perspective of liver function; however, limited researches have assessed the outcome of ACLF by adopting parameters reflecting hepatocyte regeneration. AFP is a biomarker of liver renovation; thus, we investigated the prognostic value of AFP in ACLF for that the magnitude of in the increased AFP levels is closely related to hepatocyte regeneration after acute or superimposed hepatic injury. Previous studies demonstrated that AFP was a prognostic marker in patients with acute liver failure [27, 28]. However, limited studies demonstrated a correlation between AFP levels and the outcomes of patients with ACLF, especially patients with acute hepatic failure based on chronic HBV infection.

AFP is not detected in normal adult serum. In 1963, Abelev et al. [29] found that a type of murine embryonal *α*-globulin, which was originally called AFP, could be detected in normal or malignant hepatocyte proliferation in adult rats. Then, AFP was considered a marker of hepatocyte regeneration, and the capacity of hepatocyte regeneration is key to the reversal of liver injury. The present study assessed the predictive value of AFP in the prognosis of hepatitis B-related acute-on-chronic liver failure because these patients always exhibited bad progress, and the short-term mortality is dramatically high. Valid predictive models and intensive care are necessary in the management of ACLF because some individuals regain their health following liver injury recovery and hepatocyte regeneration. Our study demonstrated that the parameter log_10_^AFP^⁡  was a helpful marker to predict the outcomes of ACLF. A higher AFP concentration could predict a better outcome of HBACLF, and log_10_^AFP^⁡  ≥ 2 could indicate a longer short-term survival time for patients with ACLF. Therefore, we speculated that the concentration of AFP may be positively related to the capacity for liver regeneration in the condition of acute liver injury on the basis of chronic liver diseases, apart from malignancies such as hepatocellular carcinoma and gastric cancer.

Consistent with Katoonizadeh's research [18], our study indicated that the difference of age was of statistical significance between survivors and those who deceased or got liver transplanted. Elderly patients exhibit a weakened condition of body function, which may result in multiple organ failure when there was a hypohepatia already. And we suspected that the number of functional hepatocytes may be lower in the elderly than young adults. In addition, total bilirubin and INR were found to be risk factors of survival as well. Previous studies demonstrated that a higher level of bilirubin was associated with a poor outcome in patients with liver failure because the dysfunctional liver exhibited deficient bilirubin metabolism, which demonstrates that the extent of damage to hepatocytes was extremely serious [8, 30]. Impeded hepatic synthetic function reduces the production of prothrombin and other proteins in the liver, which accounts for the higher level of INR, and a higher degree of INR indicates an unfavourable prognosis [31].

One limitation of our study was the lack of dynamic observations of AFP levels. A persistently low AFP may be interpreted as regenerative failure in protracted cases but ultimately leads to death. Some patients with low AFP concentrations on admission ultimately survived because liver tissue repair occurred followed with a serially intensive care. Other parameters, such as HBeAg, creatinine, albumin, and some scoring systems, could also help assess the outcome of ACLF. Therefore, further studies of multifactor-correlated prognostic models are needed.

## 5. Conclusion

In summary, AFP is an indicator of the prognosis of hepatitis B-related acute-on-chronic liver failure. Higher levels of AFP concentrations could predict a better outcome of HBACLF, and log_10_^AFP^⁡  ≥ 2 would indicate a longer survival time.

## Figures and Tables

**Table 1:** Characteristic comparisons between subgroups.

Variables	Transplant-free survival group (*n* = 40)	Deceased and transplanted group (*n* = 52)	*P* value
Age^*∗*^, y	41.83 (12.37)	50.31 (13.10)	0.002
Male *n*, %	33 (82.50%)	42 (80.77%)	0.832
Total bilirubin^#^, *μ*mol/L	261.89 (86.98–496.59)	386.55 (137.45–723.22)	<0.001
AFP^#^, ng/ml	148.80 (8.50–2375.30)	43.21 (1.10–1495.80)	<0.001
INR^#^	1.83 (1.50–4.31)	2.21 (1.51–5.70)	0.001

AFP, alpha-fetoprotein; INR, international normalized ratio; ^*∗*^normally distributed continuous variable; ^#^not normally distributed continuous variable.

**Table 2:** Univariate and multivariate Cox regression analysis for survival.

Parameter	Univariate Cox regression (*n* = 92)			Multivariate Cox regression (*n* = 92)		
	*P*	HR	CI	*P*	HR	CI
Age^*∗*^	0.003	1.033	1.011 1.056	<0.001	1.041	1.019 1.063
Total bilirubin^*∗*^	<0.001	1.004	1.002 1.006	<0.001	1.004	1.002 1.006
INR^*∗*^	0.001	1.564	1.200 2.039	0.015	1.446	1.074 1.948
log_10_^AFP#^	<0.001	2.908	1.603 5.274	0.018	2.155	1.139 4.076
Ascites^#^	0.407					
HE^#^	0.163					

AFP, alpha-fetoprotein; INR, international normalized ratio; HE, hepatic encephalopathy; HR, hazard ratio; and CI, confidence interval; ^*∗*^continuous variables and ^#^categorical variables (log_10_^AFP^ was sorted into subgroups: log_10_^AFP^ ≥ 2 and log_10_^AFP^ < 2. No statistical significance was attained between patients with or without ascites and HE).

## Data Availability

All data arising from this study are contained within the manuscript.
